# Adherence to Anti-Viral Treatment for Chronic Hepatitis B

**DOI:** 10.3390/jcm9061922

**Published:** 2020-06-19

**Authors:** Naim Abu-Freha, Muhammad Abu Tailakh, Alexander Fich, Nasreen Abu Bader, Yonat Shemer-Avni, Farhan Alsana, Nava Gasper, Heba Abu-Kaf, Ohad Etzion

**Affiliations:** 1 The Institute of Gastroenterology and Hepatology, Soroka University Medical Center, Beer-Sheva 84101, Israel; fich@bgu.ac.il (A.F.); nas.bdr112@gmail.com (N.A.B.); navagaspar@gmail.acom (N.G.); ak.heba@gmail.com (H.A.-K.); ohadet34@yahoo.com (O.E.); 2 Faculty of Health Sciences, Ben-Gurion University of the Negev, Beer-Sheva 84101, Israel; abutaila@bgu.ac.il (M.A.T.); yonat@bgu.ac.il (Y.S.-A.); 3 Recanati School for Community Health Professions, Department of Nursing, Faculty of Health Sciences, Ben-Gurion University of the Negev, Beer-Sheva 84101, Israel; 4 Nursing Research Unit, Soroka University Medical Center, Beer-Sheva 84101, Israel; 5 Virology Laboratory, Soroka University Medical Center, Beer Sheva 84101, Israel; 6 Ministry of Health, Southern Regional Office, Beer Sheva 84101, Israel; farhan.elsana@bsh.health.gov.il

**Keywords:** Hepatitis B virus, adherence, medication monitoring, Arab Bedouin, southern Israel

## Abstract

Adherence to treatment of chronic Hepatitis B Virus (HBV) is an important issue and can affect the complication rate. Nucleos(t)ide analogue as oral treatments are used for patients with necro-inflammatory activity and high viral load, with the goal of decline the complication rate such cirrhosis and hepatic cancer. We aimed to investigate the adherence to chronic HBV treatment. Chronic HBV patients with dispensing medication rates (DMR) of at least 80% were defined as high adherence group (HAG) and those who dispensed less than 80% as low adherence group (LAG). The study included 273 patients. 90 patients (33%) were in the LAG and 183 (67%) in the HAG. The All-cause mortality in the LAG was 15.6%, and 8.7% among the HAG (*p*-value = 0.09). 185 patients were of Jewish origin (mean age of 52.96 ±14.6 years, 30% women) and 88 patients of Arab Bedouin (AB) origin (mean age of 40.86 ± 13.96 years (*p*-value < 0.001), 42% women). The proportion of Jewish patients with high adherence was 71% (131 patients) versus 59% (52 patients) in AB patients (*p*-value = 0.054). The all-causes mortality was 14.6% among Jewish origin and 3.4% of AB (*p*-value = 0.01). We conclude that, two third of HBV carriers are with high level adherence to treatment in southern Israel, with lower but marginally significant all-cause mortality. No-significant differences in adherence patterns were noted between Arab Bedouin and Jews.

## 1. Background

Hepatitis B is a major global health problem. As was reported in the global Hepatitis Report of the World Health Organization, it was estimated that 257 million people were living with chronic hepatitis B virus (HBV) in 2015 [1]. The long-term sequelae cirrhosis and hepatocellular carcinoma (HCC) estimated to cause about 887,000 annual deaths [1].

Additionally, a systematic review of data published between 1965 and 2013 showed that in 2010, about 248 million individuals in the general population, worldwide were chronically infected with HBV [1,2]. 

The most feared complications of chronic hepatitis B (CHB) include cirrhosis, liver failure and hepatocellular carcinoma which accounts for a global death rate of 1 million cases annually [1,2,3,4]. The 5-year cumulative incidence of hepatocellular carcinoma was found to be 0.1%, 0.6% and 9.7% for inactive carriers, CHB patients and cirrhotic patients in Europe, and 0.3%, 2.4% and 15.5% in East Asia, respectively [5]. More advanced phases of liver disease, older age, male gender, Hepatitis B virus (HBV) genotype C and high HBV DNA viral load increase the risk for HCC devolvement [5]. 

There are several approved treatments for CHB including interferon-alpha, pegylated interferon-alfa (PegINFa) and six different nucleos(t)ide analogue (NUCs). The goal of treatment is to prevent disease progression to cirrhosis and HCC which in turn can be accomplished by inhibition of HBV replication and resolution of necro-inflammatory activity in the liver, previous studies showed that nucleos(t)ide analogue significantly reduced the risk of HCC, cirrhotic events and mortality in patients with CHB-related cirrhosis [6,7,8,9,10]. Adherence to treatment is highly important for achieving long term clinical benefit and for preventing development of resistance to NUCs.

However, maintaining longstanding adherence to anti-viral medications for HBV is challenging as most patients are asymptomatic at the time treatment is initiated and are usually committed to lifelong therapy in the absence of clear indications for treatment cessation. 

Indeed, Chotiyaputta et al., have previously reported a mean 1-year HBV treatment persistence rate of 81%, with age younger than 45 years, treatment naïve status and receipt of NUCs other than Lamivudine as factors associated with poorer adherence to treatment [11]. 

The Negev (southern region of Israel) population is comprised of a majority of Jewish inhabitants and a minority population of semi-nomad Arab Bedouins (AB). While both subpopulations have similar access to medical services, cultural and socioeconomic differences between the groups often translate into disparate health-care related outcomes. Similar to other minority populations, adherence rates to medical treatment among the AB population were found to be suboptimal. For example, 67% of hypertensive and 73% of diabetic AB patients were non-compliant with their medications despite having a higher prevalence of these diseases compared to the Jewish population [12]. 

The prevalence of HBV infection in Israel is considered to be moderate (1–3%). Approximately 100,000 people are chronically infected with the virus, with the majority being in a HBeAg negative status. Prevalence however varies considerably between different ethnic groups with higher rates reported among the Arab population [13]. Data regarding adherence to HBV therapy in Israel is scarce. The aim of the current study was to investigate factors associated with different patterns of adherence to anti-virals for HBV among infected individuals in the Negev and to compare the adherence rates between AB and Jewish patients.

## 2. Methods

### 2.1. Patients

This retrospective study included patients with CHB, who visited the outpatient liver clinic of Soroka University Medical Center (SUMC), between the years 1998 and 2015. SUMC is 1000-bed hospital, that serves as the only tertiary care center for a population of approximately 1,000,000 residents of the Negev. 

### 2.2. Data Collection

Patients’ demographic, clinical and routine laboratory data were retrieved from the computerized databases of SUMC. Data on HBV serological markers and DNA levels were obtained from the laboratory of Virology at SUMC which provides service to patients of Clalit Health Services, the largest Health Maintenance Organization (HMO) in Israel. The “at diagnosis” Laboratory values were collected during the first month of the diagnosis, while the “during treatment” values collected as the mean of all labs collected during the treatment and follow up time. 

### 2.3. Definitions

#### 2.3.1. HBV Diagnosis

Diagnosis of CHB was established on the basis of a positive HbsAg test performed on 2 separate occasions at least 6 months apart. 

#### 2.3.2. Adherence Patterns

Patients with dispensing medication rates (DMR) of 80% or more were defined as high adherence group (HAG) and those with DMR of less than 80% were categorized as low adherence group (LAG). 

#### 2.3.3. Other

Data regarding anti-viral drugs [lamivudine (LAM), adefovir (ADV), telbivudine (TBV), entecavir (ETV), tenofovir (TDF) and Peg-Interferon], including the beginning date and end date of treatment, dispensing drugs, and the number of pills dispensed to the patient during this period were collected. We compared the number of days recorded in patient care and the number of pills dispensed in order to calculate the rate of compliance of each patient. The study was carried out in accordance with the principles of the Helsinki Declaration. The study protocol was approved by the Institutional Review Board of SUMC.

### 2.4. Statistical Analysis

The results are presented as the mean±standard deviation (SD) for continuous variables and the percentage of total patients for categorical data. The statistical analysis was carried out using SPSS version 24 (IBM Corp, Armonk, NY, USA). We used student t-test for continuous variable and chi square for categorical variables, in non-parametric variables we used the Mann-Whitney U-test. A p-value less than or equal to 0.05 was considered statistically significant.

## 3. Results

During the study period, 1087 from SUMC databases were retrospectively identified as chronic HBV with chronic presence of HbsAG; of those 273 (25.1%) patients were treated for CHB and included in the analysis.

One hundred and twenty (11%) of all 1087 HBV patients were HBeAg positive, 21 (17.5%) cleared the HbeAg during the study period, but only 12 (57%) of them reached full seroconversion with appearance of HbeAb. 11 patients (52.4%) of the 21 cleared the HbsAg were treated with medications (Nucleos(t)ide Analogues-NUCs). 

### 3.1. High Versus Low Adherence Groups 

Ninety patients (33%) were in the LAG and 183 patients (67%) in the HAG. Of the HAG group, 43 patients (23.5%) displayed 100% adherence to treatment and another 67 (37%) had adherence of more than 95%. 

There were no significant differences in demographic features, background diseases or the rate of development of complications between the two groups 

The all-cause mortality was 15.6% in patients in the LAG vs. 8.7% in patients with HAG (p = 0.09) (Table 1). 

Comparison of laboratory indices of LAG vs. HAG showed, that levels of γ-glutamyl transferase (GGT), aspartate aminotransferase (AST) and alanine aminotransferase (ALT) were significantly higher at the time of diagnosis among HAG vs. LAG, these differences were no longer apparent during anti-viral treatment (Table 2).

### 3.2. Comparison of Adherence Rates between Jewish and Arab Bedouin Populations

**Figure 1:**
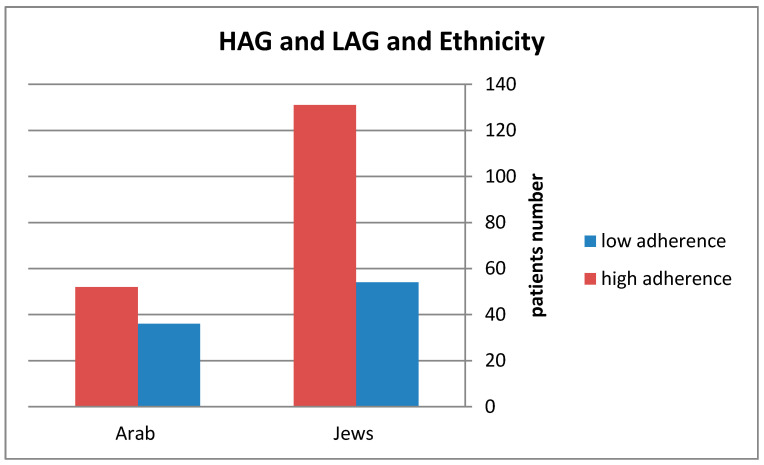
Comparison of Jews and AB patients, high and low adherence groups. HAG = High adherence group, LAG = Low adherence group.

The proportion of Jewish patients with high compliance to treatment was 71% (131 patients) versus 59% (52 patients) among AB patients with high compliance to treatment (*p*-value = 0.054). The results are summarized in Figure 1. 185 patients of Jewish origin with a mean age of 52.96 ± 14.6 years, 30% women and 88 patients of Arab Bedouin origin, a mean age of 40.86 ± 13.96 years (age difference *p*-value < 0.001), 42% women. The prevalence of diabetes among Jewish patients was 18.9%, compared with 8% among Bedouin patients (*p*-value = 0.02). 27 (14.6%) patients died (all causes mortality) of Jewish origin compared to 3 (3.4%) patients of Arab origin (*p*-value = 0.01). The demographic and clinical characteristics of the two groups are described in Table 3.

### 3.3. All Causes Mortality 

In the Jews HBV patients, 11 cases of death of the 54 patients (20.4%) with low adherence to treatment were reported and 16 from 131 (12.2%) among patients with high adherence to treatment (*p* = 0.15). 

Thirty six (41%) patients of Arab Bedouin origin were defined as low compliance, and 52 patients (59%) as high compliance. 3 patients who died (all-cause mortality) were in a group with low treatment compliance versus no deaths in the group with high compliance to treatment (*p* = 0.03). 

Focusing on the mortality causes, 25 of 30 death cases the death’s cause was reported. Among 9/25 (36%) patients of them the cause of the death was related to liver disease. The liver-related death causes were found to be the following: five (55%) patient had sepsis, two (22%) variceal bleeding, four (44%) HCC and one (11%) patient had hepatorenal syndrome (part of the patient had more than one cause of the above). 16/25 (64%) patients had liver unrelated cause of death, 13 (81.25%) patients had advanced malignancy, two (12.5%) patients had intracerebral hemorrhage and one patient (6.25%) a road accident. 

### 3.4. Medications

Our cohort of 273 patients were treated with different drugs, including combination of two drugs or switch from one medication to another, in the study period the prescribed medications were as follows: 107 (23.3%) entecavir, 53 (11.5%) adefovir, 35 (7.6%) Peg-interferon, 35 (7.6%) telbivudine, 69 (15%) tenofovir and 159 (34.7%) lamivudine.

The dispensing period for the drugs, entecavir, adefovir, telbivudine and tenofovir was between the years 2006 and 2015, however older medication includes lamivudine and interferon alpha the dispensing period was between 1998 and 2015. 

## 4. Discussion

The present study aimed to investigate the adherence rate to treatment among chronic hepatitis B carriers. In the present study, 273 (25.1%) of 1087 patients with chronic HBV were treated, the treatment rate in our study is a little higher than the treatment rate published by the World Health Organization 16.7 [1]. Pharmacy dispending data were used to determine the adherence to treatment. We found that 67% of the cohort displayed high adherence according to our pre-defined criteria (medication dispensation of at least 80% of the pills during treatment period). Our results are in agreement with previous published prospective report about adherence to entecavir [14]. However, different adherence rates for chronic HBV treatment was found in the previous published literature. On the one hand a high adherence rate of 81–99% were reported in the systemic review of Lieveld et al. [15], but on the other hand, a lower adherence rate of about 75% was found in the recently published literature, including original studies and systemic review and meta-analysis [16,17]. These differences can be accounted by the varying methods of adherence assessment used in the different studies. In our study we used the methods of computerized pills dispensing data, while other adherence assessment methods are available and used in other studies (patients self-reports, pill counts, biologic measures or electronic medical monitoring). There is no gold standard for adherence assessment method because there are advantages and disadvantages for every method.

Reliability, ease for practicing, costs and resources needed are the factors affecting the choosing the specific method for adherence assessment. In our study we choose electronic monitoring, the pill dispensing method. The advantages of this method are: computerized data, which are relative easy and not expensive to retrieved with very high accuracy of data regarding the purchased prescription. In addition, patients contact or many resources and equipment are not needed in this monitoring method. While, the disadvantage of the pill dispensing method for adherence monitoring lies in the lack of accurate data regarding the ingestion of the pills.

Our data showed that AST, ALT and GGT are significantly higher among the high adherence group at diagnosis compared with the low adherence group. This difference disappeared during treatment, most probably due to its effect on lowering viral load and diminishing necro-inflammatory activity. The majority of patients with chronic HBV infection, regardless of the severity of liver enzymes disturbances, will be asymptomatic when treatment is initiated. However, the impact of therapy will become more evident in those with higher baseline values of transaminases. Such factor may contribute to patient motivation to adhere to anti-viral therapy and may explain the increased proportion of patients with elevated liver enzymes among the HAG. 

In the present work we did not observe a significant difference between high and low adherence groups in term of comorbidities, complications, or hepatocellular carcinoma. The all-cause mortality was numerically higher among the LAG vs. HAG (15.6% vs. 8.7%), but only trended towards statistical significance. It is reasonable to assume that this difference would reach statistical significance with a higher number of patients in each group and a longer follow up period. Recently published longitudinal work found that poor adherence to medication was associated with a higher mortality and greater risk of HCC and cirrhotic complications, particularly among patients with liver cirrhosis [18].

Convincing patients with HBV to commit to lifelong therapy is challenging as the majority of the treatment eligible population will have little if any symptoms at presentation. Hepatologists and gastroenterologists managing HBV patients usually emphasize the importance of initiating anti-viral therapy for preventing future development of complications related to cirrhosis and hepatocellular carcinoma. The results of this study also underscore the importance of adhering to treatment for preventing future mortality along the disease course. 

We have compared our results according to ethnic origin, Arab Bedouin versus Jewish patients. There were non-significant differences in the proportion of HAG, between these two populations. However, AB patients included in the study are significantly younger than the included Jewish patients, with about 10 years difference, and a significantly higher frequency of diabetes mellitus and all-cause mortality were noted among the Jewish patients.

These differences reflect epidemiological trends in our region where the fast-growing Bedouin population is characterized by a younger mean age compared to the Jewish population residing in the same geographical region. This difference is strongly associated with the prevalence of metabolic diseases that become more prevalent with advancing age. 

The Bedouin population in the Negev has undergone rapid changes over the last few decades manifested by adoption of a more Westernized life style and dietary habits. We therefore expect that rates of diabetes mellitus and all-cause mortality may increase among the AB in the future, especially in the low adherence group. Previous reports showed that 67% of hypertensive and 73% of diabetic patients were not adhering to medical treatment among the Arab Bedouin [12]. We found a lower non-adhering proportion to HBV treatment (41%) compared with diabetes or hypertension in the same minority population. Some studies investigated the adherence to treatment in different diseases in minority populations, with the most important predicting factors being low education, low socioeconomic status, fear of side effects, asymptomatic disease, lack of health insurance and younger age [19,20,21,22]. 

Yoel et al., investigated the non-adherence reasons among Bedouin population in southern Israel, treated for diabetes, hypertension and dyslipidemia. Patients with low adherence to treatment were more suspicious about the prescribed drugs and tended to stop treatment at a higher rate due to perceived. In addition, low adherent patients tended to have only one disease and that was less symptomatic [23]. These patients generally exhibited poor knowledge about the complications of the chronic disease and tended to believe that drugs contain harmful chemicals [23]. The higher adherence to HBV therapy can be explained by several factors. First, most patients treated for HBV are done so by NUCs. These medications are administered once daily and are associated by few if any side effects. Second, the fear of the devastating long-term complications of HBV which include uncontrolled bleeding, cancer and premature death are probably a strong driving force for adherence to therapy. Finally, the risk of disease transmission in addition to the cultural and traditional view of infectious diseases encourage patients to maintain high adherence rates. All these factors are less relevant to other chronic disease like diabetes mellitus or hypertension. 

This study reviewed data captured between 1998 and 2015, when only about 40% of HBV patients were treated with entecavir or tenofovir. Nowadays treatment with these medications is considered first-line with only few patients treated with pegylated interferon or other NUCs. We therefore expect that adherence rates to HBV therapy in our region will increase over time. 

In summary, to the best of our knowledge this is the first study in Israel investigating the adherence to chronic hepatitis B treatment. The most important finding of our study is that CHB patients adhere to treatment in moderate level only. Adhering to CHB treatment is an important issue as the effectiveness of treatment in reducing the progression to liver disease is closely linked to adherence. The importance of the adherence were investigated by modeling studies, which showed that a lower adherence of 65% compared with a high adherence of 95% would lead to 2.6 million additional deaths over a 15-year period [24]. There is a need for a better and deep understanding of causes of nonadherence among CHB patients in order to implement intervention for nonadherence and patient’s education and assurance of the consequences including the increase risk of morbidity and mortality of being nonadherence should be a part of the patient-physician meeting. The strength of our study is the relative high number of patients, and the robustness of the database which includes information on the different treatment modalities. 

The study also presents several limitations. First, the retrospective design of the study. Second, the lack of data regarding viral load measurements before and in response to treatment for a large proportion of. Third, assessment of treatment adherence was based on to medication dispensation per computerized database. 

## 5. Conclusions

The study found that a third of chronic hepatitis B patients in our region display low adherence to treatment, with higher but marginally significant all-cause mortality. No-significant differences in adherence patterns were noted between Arab Bedouin and Jews.

## Figures and Tables

**Table 1:** Demographic and clinical characteristics of the LAG and HAG.

Characteristics	LAG *n* = 90 (%)	HAG *n* = 183 (%)	*p*-Value
Age (mean±SD) years	47.5 ± 15.3	50.2 ± 15.7	0.18
Gender female (%)	30 (33%)	63 (34%)	0.85
Days of treatment median (IQR)	617 (206, 1465)	1010 (352, 2450)	0.02
Days of follow up median (IQR)	1134 (460, 2273)	1051 (359, 2648)	0.67
DM type II (%)	18 (20)	24 (13)	0.130
Alcohol abuse (%)	2 (2.2)	3 (1.6)	0.730
Smoking (%)	13 (14.4)	27 (14.8)	0.940
HIV (%)	1 (1.1)	1 (0.5)	0.600
Drug abuse (%)	1 (1.1)	2 (1.1)	0.980
Malignancy of Liver (%)	2 (2.2)	3 (1.6)	0.730
Cirrhosis of Liver (%)	11 (12.2)	22 (12)	0.96
Hepatitis Delta (%)	1 (1.1)	2 (1.1)	0.98
Hepatic Encephalopathy (%)	4 (4.4)	5 (2.7)	0.45
Death (%)	14 (15.6)	16 (8.7)	0.090

DM = Diabetes Mellitus, HIV = Human Immunodeficiency Virus.

**Table 2:** Laboratory values of LAG and HAG at the diagnosis and during treatment.

Laboratories *		LAG *n* = 90	HAG *n* = 183	*p*-Value
**WBC**		7.52 ± 4.9	6.44 ± 2.2	0.05
**Hb**		12 ± 2.1	12.1 ± 3.3	0.97
**PLT**		189.45 ± 80	200 ± 97	0.37
**Albumin**		52.2 ± 8.13	52.4 ± 7.3	0.96
**TSH**		2.33 ± 1.44	2.3 ± 1.13	0.93
**INR**		1.24 ± 0.55	1.16 ± 0.34	0.18
**At-diagnosis**	**Alkaline Phosphatase**	98.28 ± 36.45	107.6 ± 67.7	0.16
	**Total Bilirubin**	0.988 ± 1.2	1.46 ± 2.6	0.14
	**GGT**	39.19 ± 34.6	91.41 ± 152.3	0.001
	**AST**	61.14 ± 207	174.37 ± 593	0.03
	**ALT**	73.81 ± 244.9	217.1 ± 702	0.02
	**Hepatitis HBeAg *n* (%)**	19 (28.8)	34 (22.2)	0.23
	**DNA *n* (%)** **Negative**	19 (38)	35 (26.7)	0.14
**During-treatment**	**Alkaline Phosphatase**	99.43 ± 43	96.17 ± 57.59	0.71
	**Total Bilirubin**	0.9 ± 1	0.91 ± 1.1	0.97
	**GGT**	47.32 ± 55.4	67.76 ± 133.4	0.26
	**AST**	38.3 ± 35.8	40.41 ± 50.96	0.74
	**ALT**	45.3 ± 46.41	44.41 ± 65.28	0.92
	**Hepatitis HBeAg *n* (%)**	12 (25.5)	25 (26.6)	0.89
	**DNA *n* (%)** **Negative**	25 (51)	54 (47.4)	0.67

* All values are mean ± SD, unless otherwise stated. WBC = White Blood Cells, TSH = Thyroid stimulating hormone, HB = hemoglobin, PLT = Platelets, INR=International Normalized Ratio, GGT = γ-Glutamyl transferase, AST = aspartate aminotransferase, ALT = alanine aminotransferase.

**Table 3:** Demographic and clinical characteristics of the Jews and Arab Bedouin groups.

Characteristics	Jews Patients *n* = 185	Bedouin Patients *n* = 88	*p*-Value
**Age (mean ± SD)**	52.96 ± 14.6	40.86 ± 13.96	<0.001
**Female (%)**	56 (30.3)	37 (42)	0.05
**Days of treatment median (range)**	844 (4-7218)	703 (56-5816)	0.51
**DM type II (%)**	35 (18.9)	7 (8)	0.02
**Alcohol abuse (%)**	3 (1.6)	2 (2.3)	0.700
**Smoking (%)**	30 (16.2)	10 (11.4)	0.280
**HIV (%)**	2 (1.1)	0 (0)	0.320
**Drug abuse (%)**	3 (1.6)	0 (0)	0.230
**Malignancy of liver (%)**	5 (2.7)	0 (0)	0.120
**Cirrhosis of liver (%)**	27 (14.6)	6 (6.8)	0.06
**Hepatorenal syndrome (%)**	1 (0.5)	0 (0)	0.49
**Hepatitis DELTA (%)**	3 (1.6)	0 (0)	0.23
**Portal Hypertension (%)**	9 (4.9)	4 (4.5)	0.91
**Hepatic Encephalopathy (%)**	5 (2.7)	4 (4.5)	0.45
**Death (%)**	27 (14.6)	3 (3.4)	0.01

DM = Diabetes Mellitus, HIV = Human Immunodeficiency Virus.

## Abbreviations

AB	Arab Bedouin
HBV	Hepatitis B virus
CHB	Chronic hepatitis B
DMR	Dispensing Medication Rates
HAG	High adherence group
LAG	Low adherence group
HCC	Hepatocellular Carcinoma
DM	Diabetes Mellitus
HIV	Human Immunodeficiency Virus
WBC	White Blood Cells
TSH	Thyroid stimulating hormone
HB	Hemoglobin
PLT	Platlets
INR	International Normalized Ratio
GGT	γ-Glutamyltransferase
AST	Asparatate Aminotransferase
ALT	Alanine Aminotransferase
SUMC	Soroka University Medical Center
HMO	Health Maintenance Organization
